# Development and validation of a new mpox virus sequencing and bioinformatic analysis pipeline

**DOI:** 10.1080/22221751.2025.2494733

**Published:** 2025-04-15

**Authors:** Missiani Ochwoto, Skyler Kuhn, Paul Schaughency, Ben Greene, Kailin Hawes, Felix Koukouikila-Koussounda, Reiche Golmard Elenga, Dachel Aymard Eyenet Boussam, Pembe Issamou Mayangue, Jonathan Schulz, Craig Martens, Fabien Roch Niama, Justin Lack, Ryan F. Relich, Vincent J. Munster, Claude Kwe Yinda

**Affiliations:** a Division of Intramural Research, National Institute of Allergy and Infectious Diseases, National Institutes of Health, Hamilton, MT, USA; b Research Technologies Branch, Division of Intramural Research, National Institute of Allergy and Infectious Diseases, National Institutes of Health, Hamilton, MT, USA; c Integrated Data Sciences Section, Research Technologies Branch, National Institute of Allergy and Infectious Diseases, National Institutes of Health, Bethesda, MD, USA; d Laboratoire National de Santé Publique, Brazzaville, Republic of the Congo; e Faculté des Sciences et Techniques, Marien Ngouabi University, Brazzaville, Republic of the Congo; f Department of Pathology and Laboratory Medicine, Indiana University School of Medicine, Indianapolis, IN, USA

**Keywords:** Mpox, MPXV, clades, PCR, sequencing, bioinformatics pipeline, phylogenetics

## Abstract

Sequencing and bioinformatic analysis of mpox virus (MPXV) remain challenging in resource-limited settings. We developed and validated a PCR-based sequencing assay that targets a 12.5 kilobase (kb) region that is phylogenetically representative of the whole ∼ 200 kb MPXV genome. We combined this sequencing assay with a lightweight, downloadable, on-and-off-grid-bioinformatics pipeline for rapid phylogenetic analysis. Our findings demonstrate that this simplified sequencing method, and the associated bioinformatics pipeline accurately distinguished clades, subclades, and clusters of MPXV. Therefore, this assay will provide rapid sequence information for understanding transmission patterns and sources of outbreaks in resource-limited settings. In addition, this assay provides a unique opportunity to decentralize mpox molecular surveillance capacities that are needed to contain the ongoing outbreak.

## Introduction

Mpox virus (MPXV), the cause of mpox, is a member of the *Poxviridae* family and *Orthopoxvirus* genus possessing a large linear double-stranded DNA genome ranging from 196 to 211 kb [1]. The genome encodes over 190 predicted open reading frames and its architecture is broadly categorized into three regions: a conserved central coding region flanked by variable left and right terminal regions that contain inverted terminal repeats (ITRs) [2]. These ITRs comprise identical copies of reverse complementary sequences [2].

MPXV is classified into two distinct clades, designated clade I and clade II [3]. These clades have traditionally been geographically restricted, with clade I endemic to Central Africa and clade II to West Africa. In addition, each clade is subdivided into a and b subclades [3]. Mpox manifests with lymphadenopathy and a vesiculopapular rash [4,5], with clade I associated with a higher mortality rate of approximately 3–8% [6-8] compared to 0.2–2.2% for clade II [7-9].

Historically, mpox outbreaks have been sporadic and as a result of spillover from wildlife with limited human-to-human transmission [10]. A continuous increase in mpox cases has been observed over the last two decades [11-16]. In May 2022, MPXV subclade IIb was responsible for a multinational outbreak, mainly in non-endemic regions outside West Africa [12]. Currently, there is an upsurge in clade I MPXV cases in the Democratic Republic of the Congo with subsequent spread to neighbouring countries of Burundi, Kenya, Rwanda, Uganda and Côte d’Ivoire [17] as well as countries outside the African continent such as Sweden, India, Germany and the US [18-20]. In response to the growing threat, the World Health Organization (WHO) declared mpox a Public Health Emergency of International Concern (PHEIC) twice: first on July 23, 2022, for MPXV clade II, and then on August 14, 2024, for MPXV clade I [21].

Molecular surveillance of MPXV is critical to understanding transmission patterns and evolution [22,23]. The MPXV genome's substantial size (196–211 kb) and structural complexity present significant technical and computational challenges for sequencing in resource-limited settings [24]. Here, we developed and evaluated an optimized Nanopore sequencing protocol, and an on-and-off-grid downloadable bioinformatics pipeline based on a 12.5 kb genomic region that recapitulates whole-genome evolutionary relationships.

## Methods

### Identifying a region for sequencing

**Figure 1.**
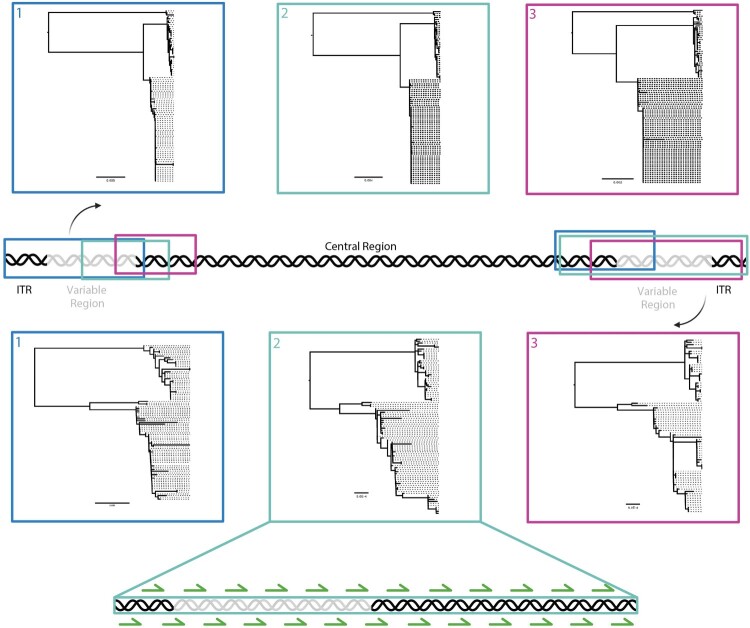
Schematic representation of the analyzed regions across the mpox virus genome. MPXV full genome sequences used for analysis were obtained from GenBank and GISAID databases and the indicated region selected for compatibility analysis: six regions of about 10–15 kb in length were selected, the top three boxes correspond to 5′ regions selected, and the bottom represents the 3′ regions selected. A set of 25 primer pairs were subsequently designed to target the most homologous region*.*

To identify the most phylogenetically representative sequence region, full genome sequences for clade I, and II from GenBank and GISAID databases were aligned using MAFFT [25]. Target loci ranging from 10 to 15 kb were selected at the 5′ and 3′ genomic termini, while regions containing sequence repeats – known to contribute minimally to phylogenetic signal – were avoided. Each selected region was aligned, and phylogenetic trees were constructed using MAFFT and IQ-TREE2 [26], respectively (Figure 1). We then evaluated the fit of each locus tree to the genome tree using IQ-TREE2’s gene concordance factor. This method assessed the support for each branch of the genome tree in the locus trees, allowing us to count the number of supporting genes. Based on this analysis, the 3′-end of the genome, encompassing all terminal repeats and a short adjacent section of the central region (Figure 1), was identified as the most representative locus for full-genome phylogeny.

### Primer design

Once the region of interest was identified, Primer Design-M [27,28] was used to generate a tiled primer pairs scheme based on a multiple sequence alignment of representative members of all MPXV clades (Figure 1). Twenty-five primer pairs were designed such that each pair amplifies a 650 - 750 nucleotide fragment. The designed primers (Supplementary Table 1) were obtained from Integrated DNA Technologies™ (IDT, USA).

### Samples and DNA extractions

Two sets of MPXV DNA samples were used in this study: MPXV cell cultured isolates and clinical specimens (Table 1). For extractions of MPXV DNA from cell culture samples, the QIAamp Viral RNA Kit (Qiagen, USA) via the QIAcube HT automated system (Qiagen, USA) was used according to the manufacturer’s instructions. The input volume was 140 µL and the elution volume was 150 µL.

**Table 1.** Characteristics of laboratory isolates and clinical samples used.

Type of sample	Sample laboratory name	Sequence name	*Lowest detection limit (copies/mL)	^#^Viral load of original sample	Ct-value	Total reads (k)
				(copies/mL)		
Laboratory isolates	hMPXV/RoC/CDC-358/2003	hMPXV-CDC-358/2003	275.25	6,631,307.5	16.4	311.1
	hMPXV-USA-2003-039	hMPXV-USA-2003-039	369.25	5,025,956.5	16.8	351.52
	hMPXV/USA/FL001/2022	hMPXV-USA/FL001/2022	246.79	4,636.9	28	86.56
Clinical samples	NA	hMPXV/USA/CLN-01/2022	N/A	2,971,649.6	17.7	391.62
	NA	hMPXV/USA/CLN-03/2022	N/A	666,396.1	20.1	380.57
	NA	hMPXV/USA/CLN-05/2023	N/A	155,287.0	22.4	249.51
	NA	hMPXV/USA/CLN-07/2023	N/A	1,249,565.2	19	46.31

*Represents the minimum number of copies detected using this method.

#Represents the viral numbers using a real time PCR. NA = Not applicable.

MPXV negative clinical samples and DNA from diagnostic MPXV PCR-positive clinical samples were obtained from the Indiana University (IU) School of Medicine. Clinical specimens were handled using enhanced-BSL-2 work practices and containment measures by vaccinated medical laboratory scientists at IU. For these samples, viral DNA was extracted using a Maxwell CSC 48 Instrument (Promega, USA), according to manufacturer’s instructions. MPXV was detected using the LightMix® Modular Orthopox Virus Typing Assay (Roche/TIB MolBiol, Germany) performed on the cobas® 4800 real-time PCR system (Roche, USA). This assay targets a 160-bp-long fragment of the RAP94 gene of orthopoxviruses using specific primers and an R6G-labeled hydrolysis probe. Following analysis, remnant DNA from MPXV-positive specimens was stored at −80°C. Each specimen was de-identified, thawed at room temperature, aliquoted, and shipped on dry ice to the Rocky Mountain Laboratories (RML) in compliance with an IU Institutional Review Board-approved protocol (IU IRB# 2004152782).

### PCR amplification

Primers were used at 10 µM. First the primers were individually optimized and then combined into five pools (A, B, C, D, and E) with each pool comprising five primer pairs. The pools were designed in a way that no consecutive primer fragments were in the same pool (Supplementary Table 1). MPXV DNA was subjected to five PCRs, amplification using primer pool A, B, C, D or E. Each PCR reaction of 25 µL included 6 µL of master mix solution (AllTaq PCR Core Kit®, Qiagen, USA), 1 µL of primer pool A, B, C, D or E, 5 µL of MPXV DNA and 13 µL of nuclease free water. The following thermal cycling conditions were used on an Eppendorf Mastercycler® X50 (Eppendorf, USA): initial denaturation at 95°C for 2 min, followed by 40 cycles at 95°C for 5 s and 50°C for 15 s, and a final extension of 72°C for 10 minutes. The presence of pooled amplified products was checked through electrophoresis on 1% agarose gel, for 40 minutes at 90 V. Visualization of the bands was done using UVP PhotoDoc-It™ systems (Analytik Jena, USA).

### Assay sensitivity

To determine the analytical sensitivity or limit of detection of the assay, a 25-fold serial dilution of viral DNA from each clade up to 7th dilution was performed. Then, each dilution was subsequently subjected to five independent PCR reactions following the primer pool scheme and analyzed via agarose gel electrophoresis, as described above.

### Assay specificity

To determine the assay specificity, we used qPCR MPXV negative clinical samples that were collected from uninfected humans or those infected either with Varicella-zoster virus (VZV) or Herpes simplex virus type 1 or 2 (HSV 1 or HSV 2). The samples were obtained from IU School of Medicine as described above. Upon arrival at RML, DNA was extracted using the QIAamp Viral RNA Kit (Qiagen, USA) via the QIAcube HT automated system (Qiagen, USA) and subjected to PCRs amplification using primer pool A, B, C, D or E as described above. The PCR products were confirmed through electrophoresis on 1% agarose gel as described above.

### Library preparation and sequencing

Equimolar amounts of each amplicon pool were mixed and subjected to end repair using NEBNext® Ultra™ II End Repair/dA-Tailing Module (New England Biolabs (NEB), USA) following manufacturer’s instructions and nCoV-2019 sequencing protocol v3 (https://www.protocols.io/view/ncov-2019-sequencing-protocol-bp2l6n26rgqe/v1, https://pubmed.ncbi.nlm.nih.gov/32908977/). Then, 3.3 µL of the pool was combined with 1.2 µL of Ultra II End Prep Reaction Buffer 0.5 µL of Ultra II End Prep Enzyme Mix, and 5 µL of nuclease free water. The reaction was incubated for 15 minutes at room temperature, 15 minutes at 65°C, and 1 min on ice. Then, 0.75 μL of the end-preparation reaction mixture were mixed with barcodes accordingly using the Oxford Nanopore native barcoding kit (EXP-NBD-104, Oxford Nanopore Technologies (ONT), UK). The reaction mix was incubated at room temperature for 20 minutes, 65°C for 10 minutes, and on ice for 1 min. Barcoded amplicons were pooled and cleaned with 0.4x volume AMPure XP beads (Beckman, USA) and eluted in 30 µL of 10 millimolar Tris HCl pH 8.0.

Sequencing adapters were ligated to the barcoded amplicons by adding 5 µL of Adapter Mix AMII (ONT, UK), 5 µL of Quick T4 DNA Ligase (NEB, USA), and 10 µL of NEBNext Quick Ligation Reaction Buffer 5x (NEB, USA) to 30 µL of the barcoded amplicon pool. Following a twenty-minute incubation at room temperature, the mix was washed with 50 µL of AMPure XP beads (Beckman, USA) and DNA eluted in 15 µL of Elution Buffer (ONT, UK). DNA was then quantified using the Qubit High Sensitivity dsDNA assay (Thermofisher, USA) following the manufacturer’s instructions.

Sequencing was performed using Oxford Nanopore’s GridION system. First, a FLO-MIN106-D flow cell was primed in accordance with the manufacturer protocol using flush tether (EXP-FLP002, ONT, UK) and flush buffer (EXP-FLP002, ONT, UK). Then 12 µL of libraries mixed with 37.5 µL of sequencing buffer (ONT, EXP-AUX001) and 25.5 µL of sequencing beads (EXP-AUX001, ONT, UK) were loaded on the flow cell and sequenced.

### MPXV bioinformatics pipeline

The OpenOmics mpox-seek [29] pipeline was developed to rapidly process targeted ONT MPXV sequencing data. The pipeline was designed to run on a standard laptop without any HPC resources or in an offline-like mode out in the field. It is highly reproducible, easy to install, and computationally lightweight.

Internally the pipeline uses Porechop [30] to perform adapter trimming. The trimmed reads are aligned against nucleotide 184133–196682 of NCBI mpox reference sequence (NC_003310.1) using minimap2 [31]. The aligned reads are sorted with samtools [32] to create a coordinate sorted BAM file. A per-sample FASTA file of viral consensus genome sequences is created with Viral Consensus [33]. The per-sample consensus sequences are concatenated and provided as input to MAFFT [25] to create a multiple sequence alignment (MSA) of the reference genome and the viral consensus sequence of each sample. From the MSA, a phylogenetic tree is built using RAxML-NG [34]. The resulting phylogenetic tree can be visualized and interactively explored with tools that can import a newick tree file. To visualize the sequencing depth along the amplified region, the pipeline produces log2 coverage plots with deepTools [35] and pyGenomeTracks [36].

### Phylogenetic analysis

All sequences obtained from the bioinformatic pipeline were manually inspected and a phylogenetic tree constructed. Alignments were built using MAFFT (FFT-NS-1 algorithm) [25], with the best model for distance estimates identified with the ModelFinder function [37] as the one with the lowest Bayesian information criterion (BIC). Maximum likelihood phylogenetic tree was constructed using IG-TREE2 [26] and branch support was assessed using both ultrafast bootstrap approximation (ufBoot, 1000 replicates) [38] and SH-like approximate likelihood ratio test (SH-aLRT). The tree was visualized in FigTree (http://tree.bio.ed.ac.uk/software/figtree/) and midpoint rooted for purpose of clarity.

## Results

### The selected region of amplification and sequencing

Targeting a 10–15 kb-long fragment, multiple regions of the MPXV genome were selected and maximum likelihood phylogenetic trees constructed (Figure 1). The best tree representative region was selected to be nucleotides 184133–196682, covering 12,549 bp (Figure 1 and Supplementary Table 2) and at least seven genes at the 3′-region of the genome using Monkeypox Virus Zaire strain (MPV-ZAI, NC-003310.1) as reference sequence [2,22].

### Targeted amplification enrichment

We developed a targeted enrichment method using a multiplex tiling PCR to amplify a ∼12.5 kb region of the MPXV genome with short amplicons (∼600–750 bp) (Figure 1). We optimized each set of the 25 primer pairs using MPXV DNA extract of known viral concentration, sequences and clades from cell culture (Table 1). All the primer sets annealed and amplified all samples in all the loci as expected (Supplementary Figure 1). We pooled the sets of primers to five pools (Pool A-E) as shown in Supplementary Table 1 and optimized using the same MPXV DNA extracts. We recorded similar results as above; all the five primer pools amplified the different loci, and the amplified fragments were one large band of 700–750 bp in a 1% agarose gel (Supplementary Figures 2–4). We conducted a sensitivity test of the optimized protocol on pooled primers and showed that we could amplify and sequence samples with genome concentrations as low as 275, 369, and 247 copies/mL, for clade I, subclade IIa and subclade IIb, respectively (Table 1, Supplementary Figure 3). We also conducted a specificity test using VZV, HSV-1 HSV-2 positive samples and negative clinical samples, all samples tested negative for all primer pools A-E (Supplementary Figure 5).

**Figure 2.**
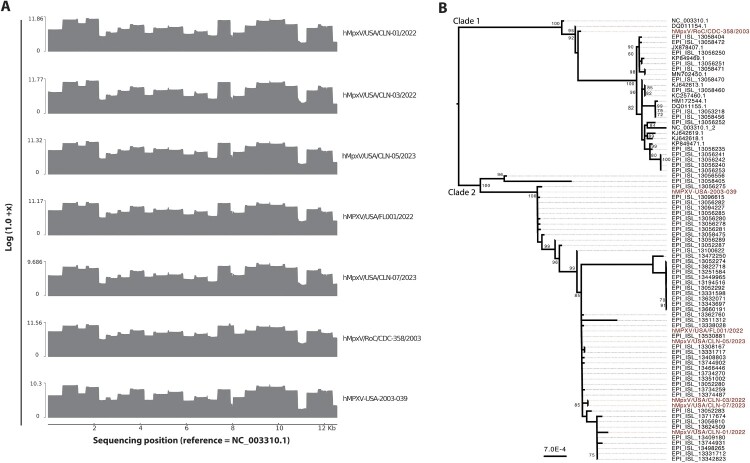
Sequencing outcome of isolates and clinical samples. (a) Schematic representation of a coverage plot across the sequenced region, x = total number of reads. (b) Maximum-likelihood tree of generated sequence isolates and clinical samples (red) and references (black). Alignments were built with MAFFT [47]. Maximum likelihood phylogenetic tree was constructed using IG-TREE2 [26] and branch support was assessed using both ultrafast bootstrap approximation (ufBoot, 1000 replicates) [38] and SH-like approximate likelihood ratio test (SH-aLRT). The tree was visualized in FigTree (http://tree.bio.ed.ac.uk/software/figtree/) and midpoint rooted. Node labels represent bootstrap values greater than 69,1,000 replicates. Scale bar indicates nucleotide substitutions per site.

To validate the method, clinical MPXV DNA samples (Ct values range 17–22) (Table 1) were used with the single and pooled primer set methods (Supplementary Figure 4). The results were similar to MPXV isolates. Sequencing on the GridION yielded reads of approximately 750 bp on average and between 10,000–100,000 reads per nucleotide position for sequences from clade I as well as subclades IIa and IIb (Figure 2(a)). When these reads were mapped to the reference locus of the genome, samples with lower Ct value resulted in higher read coverage per base (Figure 2(a), Table 1). Despite pooling equimolar amounts of each pool per sample, we observed variability in the depth of coverage across the locus for all pools and primer pairs (Figure 2(a)).

Sequences generated were analyzed via our new mpox-seek bioinformatic pipeline [29] and were subsequently used to construct a phylogenic tree including sequences of representative members of the species *Orthopoxvirus monkeypox.* The tree revealed that our sequences clustered well with clade I and II. As expected, sequences of clinical samples from mpox cases from the US clustered with subclade IIb (Figure 2(b)).

To ensure that our methods can amplify sequences from the newly described subclade Ib, we aligned our primers with the appropriate region of the sequences of this subclade and results did not show any disparity that will affect the annealing and amplification of subclade Ib. A schematic of the methods presented here is summarized in Supplementary Figure 6.

## Discussion

The WHO's PHEIC declaration for MPXV clade I underscores the urgent need to expand molecular surveillance capabilities, particularly in resource-limited settings. The implementation of robust genomic surveillance is hindered by significant infrastructural constraints, including limited access to high-throughput sequencing platforms, reagent supply chains, and computational resources required for complex bioinformatic analyses [39]. Therefore, developing streamlined sensitive, specific, and cost-effective sequencing methods coupled with simplified bioinformatic workflows is crucial for enhancing global MPXV surveillance capacity [40]. Here, we established a targeted sequencing approach using Oxford Nanopore sequencing technology combined with optimized bioinformatic analyses. The method employs a multiplexed PCR strategy comprising 25 primer pairs organized into five pools, targeting a phylogenetically informative 12.5 kb region within the 3′ terminus. Comparative phylogenetic analysis demonstrate that the topology derived from this targeted region recapitulates the evolutionary relationships observed in whole-genome phylogenies, enabling accurate clade assignment and transmission cluster identification.

To demonstrate the robustness of our method, we used clinical samples. The assay could amplify and sequence samples with viral titers as low as 246 copies/mL, which is well within range of viral loads observed in clinical samples [41]. In the ongoing mpox outbreak in the Republic of the Congo, this method was successfully used to accurately sequence samples with very low viral loads, including those with Ct values greater than 34 [13]. More recently, the same laboratory has employed this protocol to sequence samples from a clade 1b MPXV-positive case.

Analyses of the read-coverage of the amplicons revealed variation in read coverage that were consistent across different clades, suggesting different efficiencies between primer sets, but this did not affect analysis of the full MPXV sequence.

Despite Oxford Nanopore Technology's reduced infrastructural requirements and cost-effectiveness [42], bioinformatic analysis remains a significant bottleneck in genomic surveillance. Most bioinformatic tools require Linux operating systems [43], presenting a barrier in resource-limited settings where Windows OS predominates. These computational constraints often necessitate data transfer to centralized facilities with robust IT infrastructure [44], creating delays between sample collection and analysis. This lightweight, downloadable, on and off-grid bioinformatics pipeline mpox-seek has been optimized for Windows environments, requiring minimal computational resources and bioinformatics expertise to implement, hence enabling it to be the field-deployable.

The epidemiology of MPXV subclade Ib infections displays similarity with the 2022 clade IIb global outbreak, including spread via sexual networks [8,10,14,15,45,46]. However, the geographic distribution of subclade Ib as of now is still largely restricted to Africa, with laboratory confirmed high cases in resource-limited settings. This is in contrast to the subclade IIb outbreak, where the emergence and circulation of MPXV occurred undetected in Nigeria and was only recognized as a significant global health problem when it affected high-resource countries [3,12]. The complex situation of several co-circulating MPXV clades and lineages underscores the critical need for implementing accessible genomic surveillance throughout Africa. Such capacity is essential for generating real-time molecular epidemiological data to guide evidence-based public health interventions and inform policy frameworks for outbreak containment.

## Study limitation

Mpox is a zoonotic disease, although its natural animal reservoir(s) remains obscure [41]. This assay has been optimized using DNA samples derived from human MPXV infection only. Also, this assay is initially set up using Taq polymerase but can also be used with other high-fidelity polymerases to prevent PCR errors.

## Supplementary Material

Supplementary_resubmission.docx
