# Supplementary material for: Development and validation of a new mpox virus sequencing and bioinformatic analysis pipeline
Source: Emerg Microbes Infect. 2025 Apr 15;14(1):2494733. doi: 10.1080/22221751.2025.2494733 (PMC12051528; doi:10.1080/22221751.2025.2494733)
Supplement: Supplementary_resubmission.docx [file TEMI_A_2494733_SM3982.docx]

Supplementary Information for

Development and Validation of a Targeted 12.5 Kb Mpox Virus Sequencing and Bioinformatic Analysis Pipeline

**Supplementary Table 1:** Sequences of the 25 PCR primers sets and the 5 primer pools. Each primer was numbered as MPXV.FragX.FX or MPXV.FragX.RX where Frag = Fragment, X = primer number, F= Forward primer and R= Reverse primer

| **Primer name** | **Sequence 5' → 3'** | **Start** | **Stop** |
| --- | --- | --- | --- |
| MPXV.Frag1.F1 | ATGGCAACGCTACTATCCAAAC | 102 | 123 |
| MPXV.Frag1.R1 | TGAATACAATTTGGCTGTGCC | 770 | 750 |
| MPXV.Frag2.F2 | GTAGGTGATTACGGCACAGCC | 738 | 758 |
| MPXV.Frag2.R2 | CTAAGCTAGATTCACACTCATGATC | 1413 | 1389 |
| MPXV.Frag3.F3 | TAGTCAGGATTATGGAGATGGTAC | 1334 | 1357 |
| MPXV.Frag3.R3 | ATCTGTTTATAGACGCCGGC | 1890 | 1871 |
| MPXV.Frag4.F4 | CCTCCTTACACATTCGAATTC | 1746 | 1766 |
| MPXV.Frag4.R4 | CTTTGGCGTCTTTTGTATCC | 2322 | 2303 |
| MPXV.Frag5.F5 | TAACAAATCCTATGTGTTAGTAAAC | 2198 | 2222 |
| MPXV.Frag5.R5 | TTTAGAAGACTTATTCGGAAAG | 2828 | 2807 |
| MPXV.Frag6.F6 | GAAACAGTGTCTATGGAAATTATTG | 2616 | 2640 |
| MPXV.Frag6.R6 | AGAGAGACAGTCTCCTACTACAG | 3280 | 3258 |
| MPXV.Frag7.F7 | CGATAAAGTACAACATCAAGAC | 3125 | 3146 |
| MPXV.Frag7.R7 | TTACCATCAGAAGCTTTAAGGAG | 3804 | 3782 |
| MPXV.Frag8.F8 | ATTGTCCAAGTGACACATTCAC | 3659 | 3680 |
| MPXV.Frag8.R8 | CAAAGTATAAAAATACTGCAGCTG | 4292 | 4269 |
| MPXV.Frag9.F9 | CATACGAATGGCATTTCTAAG | 4202 | 4222 |
| MPXV.Frag9.R9 | TTAATTTCCATTTTTAATGGTGTCG | 4800 | 4776 |
| MPXV.Frag10.F10 | TGGATAGAAACCAAGGTTGTTAG | 4676 | 4698 |
| MPXV.Frag10.R10 | GAGTATTAGTGAGAGAGCATG | 5355 | 5335 |
| MPXV.Frag11.F11 | CATATCCTCACTGCTAAATAC | 5303 | 5323 |
| MPXV.Frag11.R11 | TGTGGGATTGTAGTTTACTTAG | 5978 | 5957 |
| MPXV.Frag12.F12 | CTATATTCCTACTAGTTTGCTGC | 5884 | 5906 |
| MPXV.Frag12.R12 | TTATACAATCTTCGCTATCGAACC | 6546 | 6523 |
| MPXV.Frag13.F13 | TTGATAGATCGAAAAAAGCCAC | 6454 | 6475 |
| MPXV.Frag13.R13 | AATATCTTCCATATAATTCTTTGAC | 7038 | 7014 |
| MPXV.Frag14.F14 | TTATATCTGCATTAGTGTCAAAG | 6998 | 7020 |
| MPXV.Frag14.R14 | AAAATACTTGATACAGAGCTTTATC | 7662 | 7638 |
| MPXV.Frag15.F15 | GTCAAC**G**CAGTAATGAGTAATGG | 7462 | 7484 |
| MPXV.Frag15.R15 | AGCTTGTGAAACAATTAGATCCC | 8028 | 8006 |
| MPXV.Frag16.F16 | TAAACAATGTATCAACTGATGAC | 7952 | 7974 |
| MPXV.Frag16.R16 | ATGATTTACTTTTTCATAATTAGAG | 8628 | 8604 |
| MPXV.Frag17.F17 | CACTTCTAATTTTTATACCTTGATC | 8574 | 8598 |
| MPXV.Frag17.R17 | GTTTACAGTAGTAATGAAGAGAAG | 9192 | 9169 |
| MPXV.Frag18.F18 | GGAGCTTAAACTATTACGTTCTG | 9091 | 9113 |
| MPXV.Frag18.R18 | TTGTTATTGCAATTCTGGAAG | 9697 | 9677 |
| MPXV.Frag19.F19 | AGTCGCTCTCTCAATATACTACG | 9585 | 9607 |
| MPXV.Frag19.R19 | TACATGGGAAATGAAATGCGTTTG | 10192 | 10169 |
| MPXV.Frag20.F20 | CCATACATTCAGACAAACGC | 10156 | 10175 |
| MPXV.Frag20.R20 | AGACAATAATATCCTGGAGAGC | 10842 | 10821 |
| MPXV.Frag21.F21 | TTTACAGGCTTGTCTAAGTTGTAAC | 10729 | 10753 |
| MPXV.Frag21.R21 | TGTC**GA**CGCTAGATAGACAGTCG | 11387 | 11365 |
| MPXV.Frag22.F22 | TAACGAAAACAAAGAATGATAC | 11307 | 11328 |
| MPXV.Frag22.R22 | TCTCTGTAACTTCCGTTACG | 11869 | 11850 |
| MPXV.Frag23.F23 | AAAACAAACATCATATGGGAATC | 11765 | 11787 |
| MPXV.Frag23.R23 | ACATGCTTTGAGTTTTGTTGAATC | 12423 | 12400 |
| MPXV.Frag24.F24 | AGAGTTTAGCGTACGTATCGG | 12279 | 12299 |
| MPXV.Frag24.R24 | TCCCCTACATGGATTTTACAG | 12934 | 12914 |
| MPXV.Frag25.F25 | ACATCATATAATAAACGTATATAG | 12865 | 12888 |
| MPXV.Frag25.R25 | AAAGGATAAAAACTTTTTAC | 13133 | 13114 |
| **Primer pool A** – Fragment sets. 1, 4, 7, 10 and 13,  **Primer pool B** – Fragment sets. 2, 5, 8, 11 and 14,  **Primer pool C** – Fragment sets. 3, 6, 9, 12 and 15.  **Primer pool D** – Fragment sets. 16, 18, 20, 22 and 24.  **Primer** **pool E** – Fragment sets. 17,19, 21, 23 and 25 | | | |

**Supplementary Table 2:** Evaluation of each locus tree to the genome using IQ-TREE2’s gene concordance factor.

| **ID** | 5' region 1 | 5' region 2 | 5' region 3 | 3' region 1 | 3' region 2 | 3' region 3 |
| --- | --- | --- | --- | --- | --- | --- |
| **Branches supported** | 23 | 23 | 23 | 16 | 21 | 24 |
| 94 | 0 | 1 | 1 | 1 | NA | 1 |
| 95 | 1 | 1 | 1 | 1 | 1 | 1 |
| 96 | 1 | 1 | 1 | 1 | 1 | 1 |
| 97 | 0 | 0 | 1 | 0 | 1 | 0 |
| 98 | 1 | 0 | 0 | 0 | 1 | 1 |
| 99 | 1 | 1 | 1 | 0 | 1 | 0 |
| 100 | 1 | 0 | 0 | 1 | 1 | 1 |
| 101 | 1 | 1 | 1 | 1 | 0 | 1 |
| 102 | 1 | 1 | 1 | 1 | 1 | 1 |
| 103 | 1 | 1 | 1 | 1 | 1 | 1 |
| 104 | 1 | 1 | 1 | 1 | 1 | 1 |
| 105 | 1 | 0 | 1 | 0 | 1 | 1 |
| 106 | 1 | 1 | 1 | 1 | 1 | 1 |
| 107 | 0 | 0 | 0 | 0 | 0 | 0 |
| 108 | 0 | 0 | 0 | 0 | 0 | 0 |
| 109 | 0 | 0 | 1 | 0 | 0 | 0 |
| 110 | 0 | 0 | 0 | 0 | 0 | 0 |
| 111 | 0 | 0 | 0 | 0 | 0 | 0 |
| 112 | 0 | 0 | 0 | 1 | 1 | 1 |
| 113 | 0 | 0 | 0 | 0 | NA | 0 |
| 114 | 1 | 1 | 1 | 1 | NA | 1 |
| 115 | 1 | 0 | 0 | 0 | NA | 0 |
| 116 | 1 | 0 | 0 | 0 | NA | 0 |
| 117 | 0 | 0 | 0 | 0 | 1 | 0 |
| 118 | 0 | 1 | 1 | 0 | 0 | 0 |
| 119 | 1 | 1 | 0 | 0 | 0 | 0 |
| 120 | 1 | 1 | 0 | 1 | 0 | 1 |
| 121 | 1 | 1 | 1 | 1 | 1 | 1 |
| 122 | 0 | 0 | 0 | 0 | 0 | 0 |
| 123 | 1 | 1 | 0 | 0 | 1 | 1 |
| 124 | 1 | 1 | 1 | 0 | NA | 1 |
| 125 | 1 | 1 | 1 | 0 | 1 | 1 |
| 126 | NA | 1 | 1 | 1 | 1 | 1 |
| 127 | NA | NA | NA | 1 | NA | 1 |
| 128 | 1 | 1 | 1 | 1 | 1 | 1 |
| 129 | 0 | 0 | 0 | 0 | 1 | 0 |
| 130 | 0 | 0 | 0 | 0 | 0 | 0 |
| 131 | 0 | 0 | 0 | 0 | 0 | 0 |
| 132 | 0 | 0 | 0 | 0 | 0 | 0 |
| 133 | 0 | 0 | 0 | 0 | 0 | 0 |
| 134 | 0 | 0 | 0 | 0 | NA | 0 |
| 135 | 0 | 0 | 0 | 0 | NA | 0 |
| 136 | 0 | 0 | 1 | 0 | 0 | 0 |
| 137 | 0 | 1 | 1 | 0 | 0 | 0 |
| 138 | 0 | 0 | 0 | 0 | NA | 0 |
| 139 | 0 | 0 | 0 | 0 | 0 | 0 |
| 140 | 0 | 0 | 0 | 0 | NA | 0 |
| 141 | 0 | 0 | 0 | 0 | NA | 1 |
| 142 | 0 | 0 | 1 | 0 | NA | 0 |
| 143 | 1 | 1 | 1 | 0 | NA | 0 |
| 144 | 0 | 0 | 0 | 0 | 0 | 0 |
| 145 | 0 | 0 | 0 | 0 | 0 | 0 |
| 146 | 0 | 0 | 0 | 0 | 0 | 0 |
| 147 | 0 | 0 | 0 | 0 | 0 | 0 |
| 148 | 0 | 0 | 0 | 0 | 0 | 0 |
| 149 | 0 | 0 | 0 | 0 | 0 | 0 |
| 150 | 0 | 0 | 0 | 0 | 0 | 0 |
| 151 | 0 | 0 | 0 | 0 | 0 | 0 |
| 152 | 0 | 0 | 0 | 0 | 0 | 0 |
| 153 | 0 | 0 | 0 | 0 | 0 | 0 |
| 154 | 0 | 0 | 0 | 0 | 0 | 0 |
| 155 | 0 | 0 | 0 | 0 | 0 | 0 |
| 156 | 1 | 1 | 0 | 0 | 1 | 1 |
| 157 | 0 | 0 | 0 | 0 | 0 | 0 |
| 158 | 0 | 0 | 0 | 0 | 0 | 0 |
| 159 | 0 | 0 | 0 | 0 | 0 | 0 |
| 160 | 0 | 0 | 0 | 0 | 0 | 0 |
| 161 | 0 | 0 | 0 | 0 | 0 | 0 |
| 162 | 0 | 0 | 0 | 0 | 0 | 0 |
| 163 | 0 | 0 | 0 | 0 | 0 | 0 |
| 164 | 0 | 0 | 0 | 0 | 0 | 0 |
| 165 | 0 | 0 | 0 | 0 | 0 | 0 |
| 166 | 0 | 0 | 0 | 0 | 0 | 0 |
| 167 | 0 | 0 | 0 | 0 | 0 | 0 |
| 168 | 0 | 0 | 0 | 0 | 0 | 0 |
| 169 | 0 | 0 | 0 | 0 | 0 | 0 |
| 170 | 0 | 0 | 0 | 0 | 0 | 0 |
| 171 | 0 | 0 | 0 | 0 | 0 | 0 |
| 172 | 0 | 0 | 0 | 0 | 0 | 0 |
| 173 | 0 | 0 | 0 | 0 | 0 | 0 |
| 174 | 0 | 0 | 0 | 0 | 0 | 0 |
| 175 | 0 | 0 | 0 | 0 | 0 | 0 |
| 176 | 0 | 0 | 0 | 0 | 0 | 0 |
| 177 | 0 | 0 | 0 | 0 | 0 | 0 |
| 178 | 0 | 0 | 0 | 0 | 0 | 0 |
| 179 | 0 | 1 | 0 | 0 | NA | 0 |
| 180 | 0 | 0 | 0 | 0 | NA | 0 |
| 181 | 0 | 0 | 0 | 0 | 0 | 0 |
| 182 | 0 | 0 | 0 | 0 | 1 | 0 |
| 183 | 0 | 0 | 0 | 0 | 0 | 1 |
|  |  |  |  |  |  |  |

0 = indicated the locus was not fit.

1 = Indicated the locus tree was fit to the genome tree and

NA = not applicable

We selected a locus that had the highest number of fit to the genome that is 3' region 3 locus.


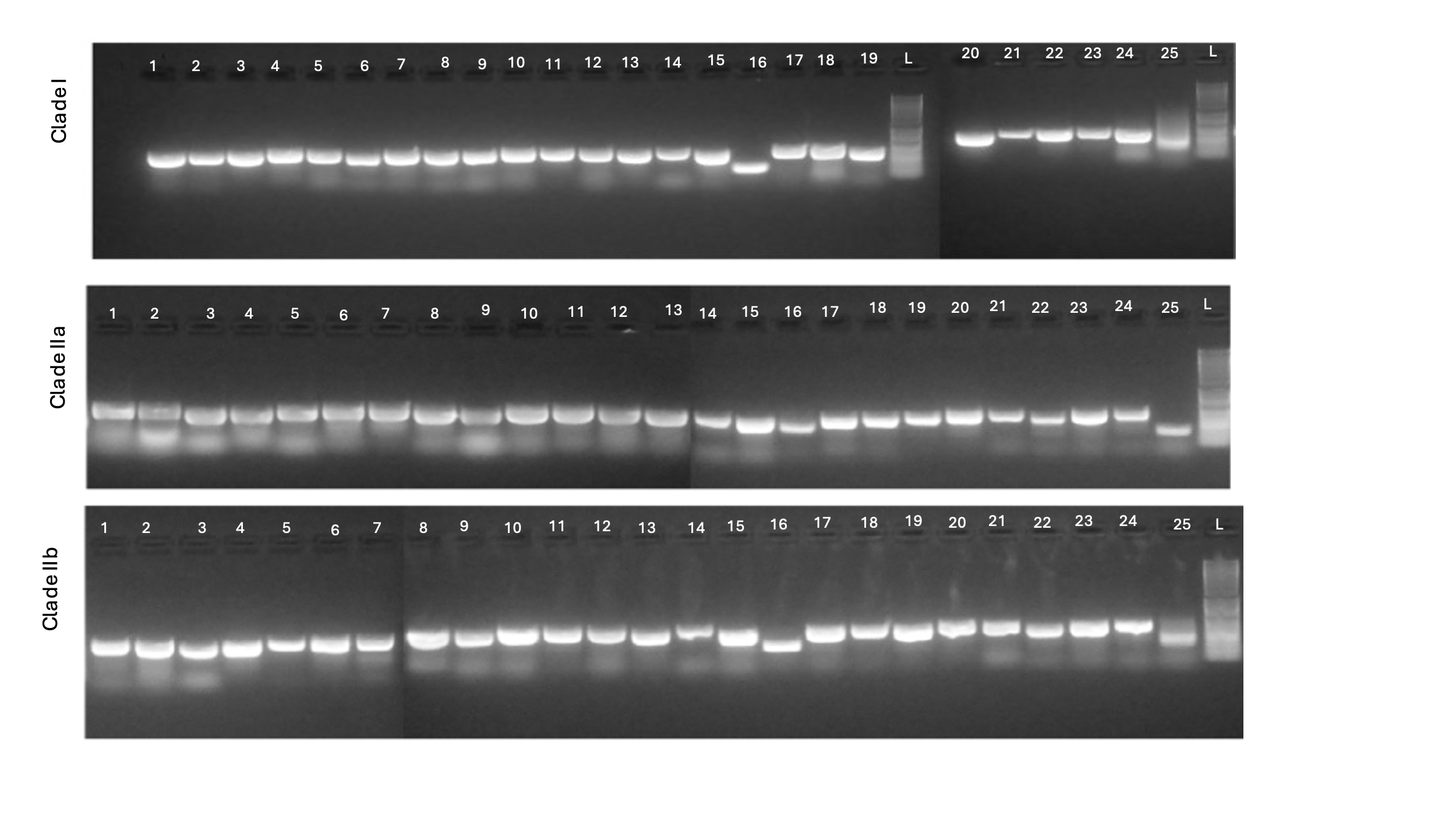


**Supplementary Figure 1:** Gel electrophoresis of the 25 primer sets listed as 1-25. The primer sets amplified all the loci of DNA isolates from MPXV clades I, IIa and IIb. The numbers (1-25) represent individual primer sets (refer to supplementary table 1).


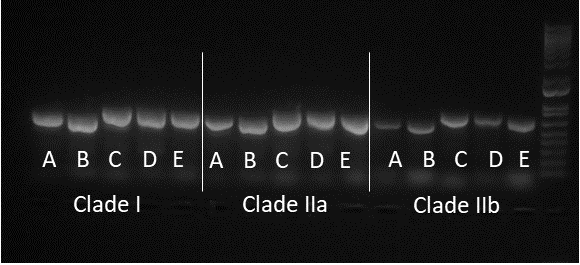


**Supplementary Figure 2:** A 1% agarose gel electrophoresis showing primer pools A, B, C, D, and E (Refer to supplementary table 2 for details). The primer pools were optimized, and we observed that they were able to amplify samples from different clades and subclades. Samples clade 1 is hMpxV/Republic of the Congo/CDC-358/2003, Clade IIa is hMPXV-USA-2003-039 and Clade IIb is hMPXV/USA/FL001/2022.


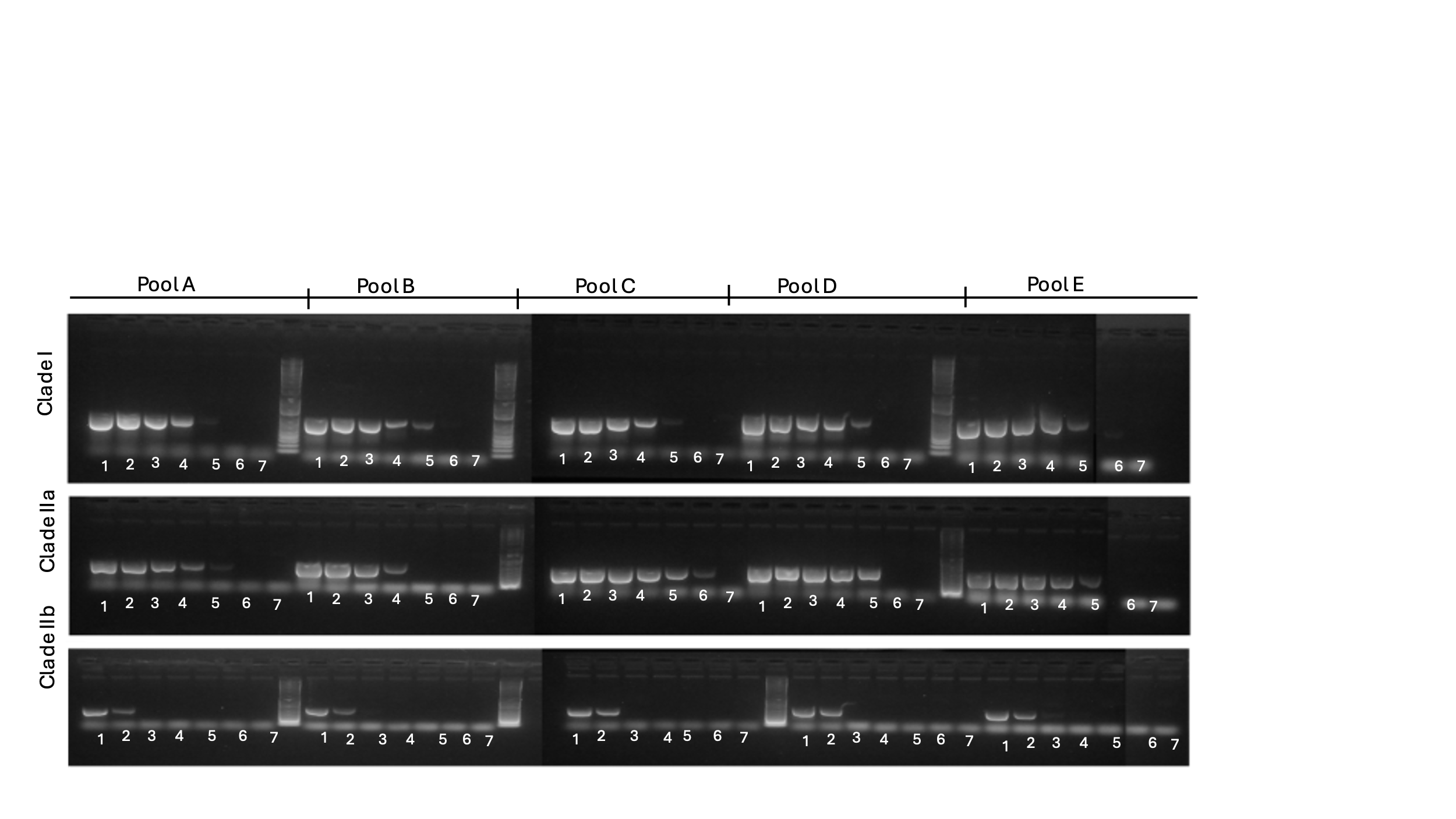
**Supplementary Figure 3:** Ethidium bromide-stained PCR products visualized after electrophoresis in 1X TAE buffer on a 1% agarose gel using tissue culture virus isolate dilution (stock virus load, clades I =2,950,000 copies/mL; clades IIa=3,630,000 copies/mL; clades IIb=2,330,000 copies/mL). Refer to Supplement Table 2 for dilution scheme.


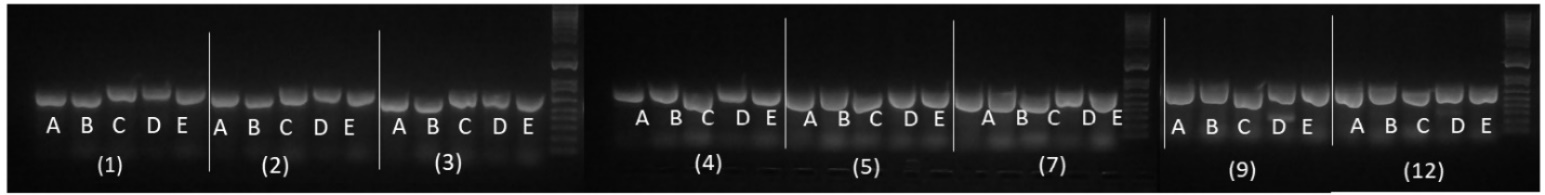


**Supplementary Figure 4:** Image of 1% agarose gel electrophoresis of the clinical samples amplified using primer pools A, B, C, D, and E (Refer to supplementary table 2 for details). The clinical samples (1) = hMPXV/USA/CLN-01/20XX, (2)= hMPXV/USA/CLN -02/20XX, (3) = hMPXV/USA/CLN -03/20XX, (4) = hMPXV/USA/CLN -04/20XX, (4)= hMPXV/USA/CLN -05/20XX, (7) = hMPXV/USA/CLN -07/20XX, (9) = hMPXV/USA/CLN -09/20XX and (12) = hMPXV/USA/CLN -12/20XX. The optimized primer pools were able to amplify the clinical samples in the same manner as the laboratory isolates


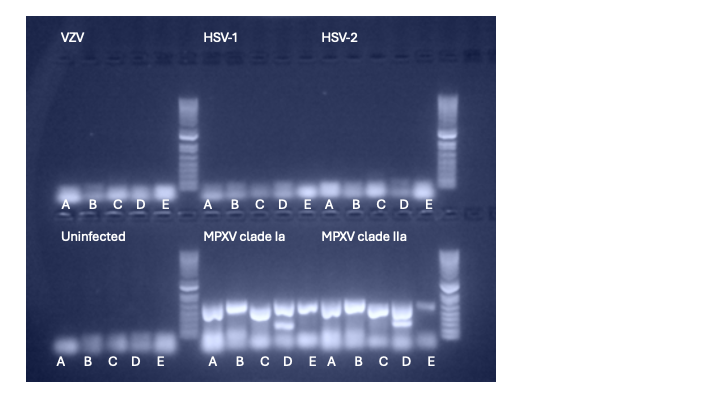


**Supplementary Figure 5:** Image of 1% agarose gel electrophoresis of Mpox negative clinical samples using primer pools A, B, C, D and E. The clinical samples were from positive cases of VZV (Varicella-zoster virus), HSV 1 (Herpes simplex virus type 1), and HSV 2 (Herpes simplex virus type 2), and patient sample negative for all these pathogens. Positive controls are MPXV clade Ia and IIa


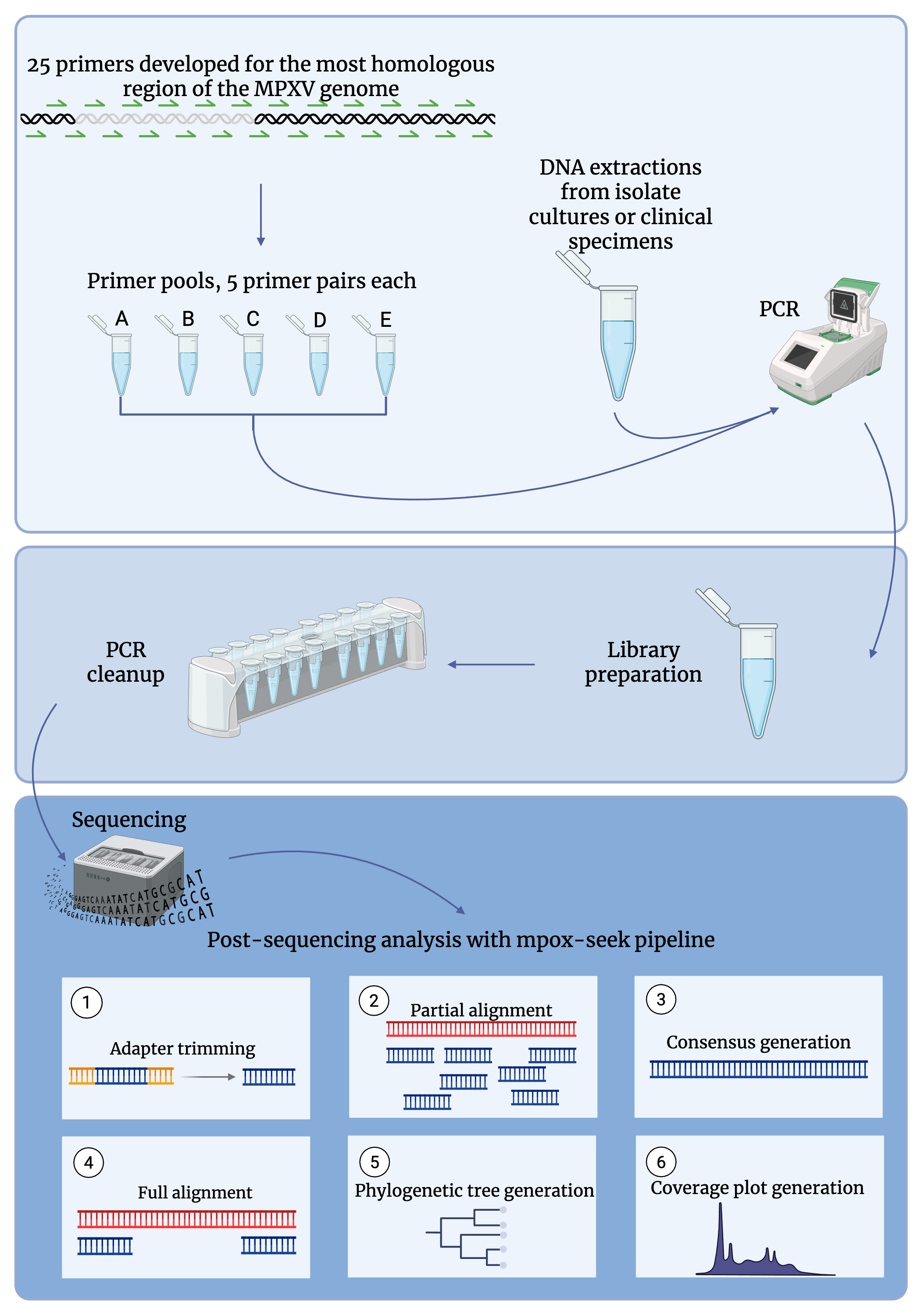


**Supplementary Figure 6:** A schematic of the methodology presented in the paper. Figure was generated using Biorender (https://app.biorender.com/illustrations/)
